# Cell-Free Circulating Mitochondrial DNA Levels Following High-Frequency Jet Ventilation—A Post Hoc Analysis

**DOI:** 10.3390/jcm14186528

**Published:** 2025-09-17

**Authors:** Marita Windpassinger, Michal Prusak, Kurt Ruetzler, Olga Plattner, Isabella Stanisz, Patrick Haider

**Affiliations:** 1 Department of Anesthesia, Critical Care and Pain Medicine, Division of General Anesthesia and Intensive Care Medicine, Medical University of Vienna, 1090 Vienna, Austria; michal.prusak@meduniwien.ac.at (M.P.); olga.plattner@meduniwien.ac.at (O.P.); 2 Outcome Research Consortium, Houston, TX 77030, USA; kurt.ruetzler@ordensklinikum.at; 3 Department of Anesthesiology and Intensive Care Medicine, Ordensklinikum Linz, 4020 Linz, Austria; 4 Department of Otorhinolaryngology, Medical University of Vienna, 1090 Vienna, Austria; isabella.stanisz@meduniwien.ac.at; 5 Department of Internal Medicine II, Medical University of Vienna, 1090 Vienna, Austria; patrick.haider@meduniwien.ac.at

**Keywords:** cell-free circulating mitochondrial DNA, high-frequency jet ventilation, oxidative stress, anesthesia

## Abstract

**Background**: Mitochondrial DNA (mtDNA), normally enclosed within mitochondria, can be released into circulation in response to cellular stress, hypoxia, or inflammation. Its detection in plasma has been proposed as a marker of cellular injury, particularly in the context of mechanical ventilation. High-frequency jet ventilation is a specialized approach of open-airway ventilation, delivering small tidal volumes through jet gas streams, applied with high pressure and oxygen fraction. It remains unclear whether this mode of ventilation contributes to mitochondrial stress. We therefore hypothesized that circulating mtDNA levels would increase after jet ventilation due to the combined effects of high oxygen exposure and mechanical strain. Furthermore, we explored whether the magnitude of mtDNA change correlates with the duration of ventilation and arterial oxygenation levels. **Methods**: Plasma levels of cell-free circulating mitochondrial DNA were measured in 30 patients before and following jet ventilation in laryngotracheal surgery. Post hoc analysis of a primary monocentric, randomized cross-over study was conducted to investigate ventilation distribution in high-frequency jet ventilation techniques. **Results:** Mitochondrial DNA levels significantly decreased after jet ventilation (median T0: 13.57; T1: 6.78; *p* = 0.0087). No significant associations were found between mtDNA change and jet ventilation duration, type of surgery, or ASA classification. Despite variable air entrainment in the open-jet ventilation system, the arterial partial pressure of oxygen increased significantly during the procedure. **Conclusions**: Jet ventilation was associated with a significant decrease in circulating mtDNA levels. This contrasts with our initial hypothesis of mtDNA elevation under ventilation-induced stress. These findings suggest that jet ventilation may exert less mitochondrial damage than previously expected.

## 1. Introduction

Standard intraoperative monitoring does not reliably detect early molecular signs of ventilation-induced cellular strain. Several studies have shown that even short periods of mechanical ventilation can trigger lung inflammation and epithelial damage [1,2,3]. These findings support the use of molecular markers, such as circulating mitochondrial DNA (mtDNA), to detect early, subclinical cellular injury. MtDNA, which encodes key components of the respiratory chain, can be released into circulation during necrosis or oxidative stress, as may occur during mechanical ventilation [4,5].

High inspiratory oxygen concentrations, as commonly used during jet ventilation, may lead to hyperoxemia (PaO_2_ > 100 mmHg), which promotes the generation of reactive oxygen species (ROS) and oxidative stress in alveolar epithelial cells [1,6,7]. This oxidative burden can impair mitochondrial integrity and trigger the release of mtDNA into circulation [6,8,9]. Although this mechanism has been described in conventional ventilation and hyperoxic models [10], its relevance in high-frequency open ventilation techniques remains unclear.

Surgical procedures involving the larynx and trachea present unique challenges due to the shared airway between anesthesiologists and surgeons and the limited access for conventional ventilation techniques via endotracheal tube. In cases where an unobstructed view of the surgical field is essential, the use of a conventional endotracheal tube is unfeasible. High-frequency ventilation techniques, such as jet ventilation, are among the methods used to maintain oxygenation in these settings [11,12,13,14]. Jet ventilation delivers small tidal volumes at high frequency and driving pressure via an open airway system, making real-time tidal volume monitoring unfeasible [15,16]. Despite its routine clinical use, its impact on pulmonary cellular integrity has not been thoroughly investigated. Research on ventilator-induced lung injury (VILI) has focused on conventional ventilation strategies, with circulating mtDNA levels explored as a marker of lung tissue damage [5,17,18,19].

Whether open, high-frequency ventilation techniques, such as jet ventilation, together with the high oxygen concentration they require, contribute to mitochondrial damage or mtDNA release remains unclear. While prior studies have demonstrated increased mtDNA levels under conventional mechanical ventilation and hyperoxic conditions, it is unclear whether this also applies to open-system strategies such as jet ventilation. While experimental data link hyperoxia to mitochondrial damage and mtDNA release, evidence specific to open-system ventilation is limited [7,20]. We therefore hypothesized that circulating mtDNA levels would increase after jet ventilation due to the combined effects of high oxygen exposure and mechanical strain. Furthermore, we explored whether the magnitude of mtDNA change correlates with the duration of ventilation and arterial oxygenation levels. To address this, the present study quantified plasma mtDNA concentration before and after jet ventilation and examined its relationship with the duration of jet ventilation and gas exchange parameters from arterial blood gases. Blood samples were collected from a cohort of patients enrolled in a previously conducted clinical study investigating the shift in ventilation distribution during jet ventilation [21].

## 2. Materials and Methods

This post hoc analysis is based on data from a previously conducted randomized cross-over trial, which received approval from the local ethics committee of the Medical University of Vienna, Vienna, Austria (EC No. 1298/2019; 9 April 2019). The trial was registered on ClinicalTrials.gov by Marita Windpassinger on 4 June 2019 (ClinicalTrials.gov Identifier NCT03973294) [21]. All participating patients provided written informed consent prior to inclusion.

### 2.1. Subject Selection

Briefly, the original study included 30 adult patients (≥18 years) scheduled for laryngotracheal surgery requiring jet ventilation. Its primary aim was to assess the shift in ventilation distribution across the lung cross-section under jet ventilation. For the current analysis, we focused on plasma mtDNA levels measured before and after jet ventilation, in order to investigate potential perioperative changes associated with this specific ventilation technique.

### 2.2. Protocol

The protocol of the original study included data collection during elective laryngotracheal surgery under jet ventilation, including perioperative blood sampling (ClinicalTrials.gov Identifier NCT03973294) [21]. The present mtDNA evaluation was performed post hoc using residual blood material and constitutes a secondary post hoc exploratory analysis.

### 2.3. Measurements

Patients who underwent laryngotracheal surgery received jet ventilation. Arterial blood samples were collected immediately before the induction of anesthesia and at the end of jet ventilation. The samples were centrifuged at 4 °C at 2000 rpm for 15 min and subsequently stored at −80 °C until analysis of mtDNA levels.

Quantification of circulating mtDNA was performed using quantitative polymerase chain reaction (pPCR) as described before [22]. Plasma samples were centrifuged at 18,000 g at 4 °C for 15 min. DNA extraction was carried out using the Maxwell 16 Robot, together with a Maxwell 16 Blood DNA Purification Kit (Promega, Madison, WI, USA). Plasma DNA copy numbers were determined by qPCR using the Absolute Human Mitochondrial DNA Copy Number Quantification qPCR Assay Kit (ScienCell Research Laboratories, Carlsbad, CA, USA) according to the manufacturer’s instructions, which detects the most conserved human mtDNA regions. Thermal cycling conditions included an initial denaturation at 95 °C for 10 min, followed by 32 amplification cycles of 95 °C for 20 s, 52 °C for 20 s, and 72 °C for 45 s; qPCR was performed on a Bio-Rad CFX384 Real-Time System (Watford, UK). Using the provided reference human genomic DNA samples and SCR primers, individual copy numbers for each sample were calculated using the Ct method.

### 2.4. Statistical Methods

The main outcome parameter was the change in circulating mtDNA copy number (detectable mtDNA fragments per volume plasma) between baseline prior to anesthesia (T0) and post-jet ventilation (T1), assessed using the Wilcoxon signed-rank test.

Secondary outcomes included associations between the change in mtDNA levels (ΔmtDNA) and the duration of jet ventilation (analyzed using the Spearman rank correlation), as well as type of surgical intervention (Kruskal–Wallis test), and changes in arterial gas exchange parameters, including Δ in arterial partial pressure of oxygen (ΔPaO_2_), Δ arterial oxygen saturation (ΔSaO_2_), Δ arterial partial pressure of carbon dioxide (ΔPaCO_2_), and Δ hemoglobin (ΔHb), analyzed using Spearman rank correlation.

Potential associations between ΔmtDNA or baseline mtDNA levels and demographic variables were explored using point-biserial correlation for sex, Spearman rank correlation for age, and the Kruskal–Wallis test for ASA classification.

Data were analyzed using IBM SPSS Statistics (Version 28, IBM Corp., Armonk, NY, USA). A *p*-value < 0.05 was considered statistically significant. Given the hypothesis-generating nature of this study, all secondary and subgroup analyses were interpreted in an exploratory manner. No adjustments for multiple comparisons were applied.

## 3. Results

A total of 30 patients were included, with a mean age of 48.6 years (SD ± 16.4; range: 22–79 years). Forty percent of the cohort was female, and the average body weight was 77.2 kg (SD ± 12.0). The majority of patients were classified as ASA I (*n* = 19), while nine were ASA II and two were ASA III (Table 1).

**Figure 1:**
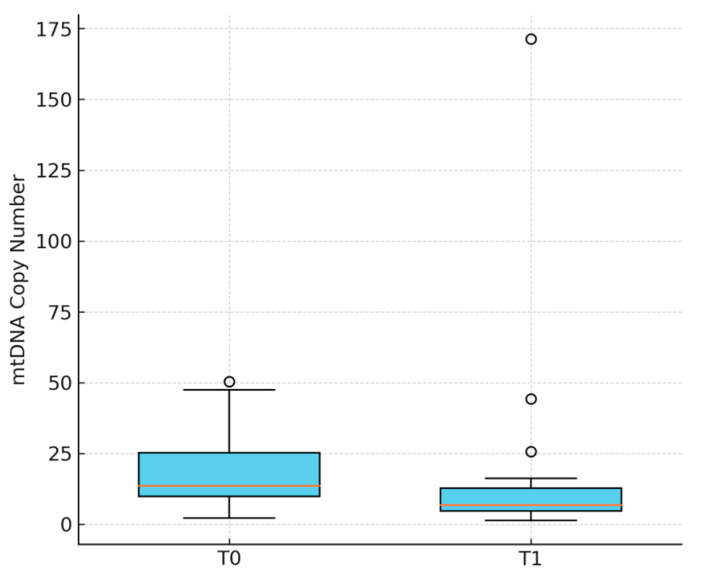
Boxplot of mtDNA levels (copy number) at baseline (T0) and after jet ventilation (T1). Boxplot comparing mtDNA levels at both time points. Each box shows the distribution of values across participants, including median and interquartile range. A decrease in mtDNA levels from T0 to T1 supports the statistical result obtained via the Wilcoxon signed-rank test (*p* = 0.0087).

Circulating mtDNA levels declined significantly following jet ventilation (ΔmtDNA; median 13.57 vs. 6.78, *p* = 0.0087; Wilcoxon signed-rank test; see Figure 1).

There were no statistically significant associations between ΔmtDNA and the duration of jet ventilation (range: 27–172 min, median 61 min, *ρ* = 0.24, *p* = 0.20) or the type of surgical procedure (*H* = 5.94, *p* = 0.20, Kruskal–Wallis test).

The median arterial partial pressure of oxygen (PaO_2_) and arterial oxygen saturation (SaO_2_) increased from baseline to post-jet ventilation (PaO_2_, 89.3 mmHg vs. 205.0 mmHg, *p* < 0.001; SaO_2_, 96.9% vs. 99.4%, *p* < 0.001). Arterial partial pressure of carbon dioxide (PaCO_2_) and changes in hemoglobin concentration (Hb) are presented in Table 2.

**Figure 2:**
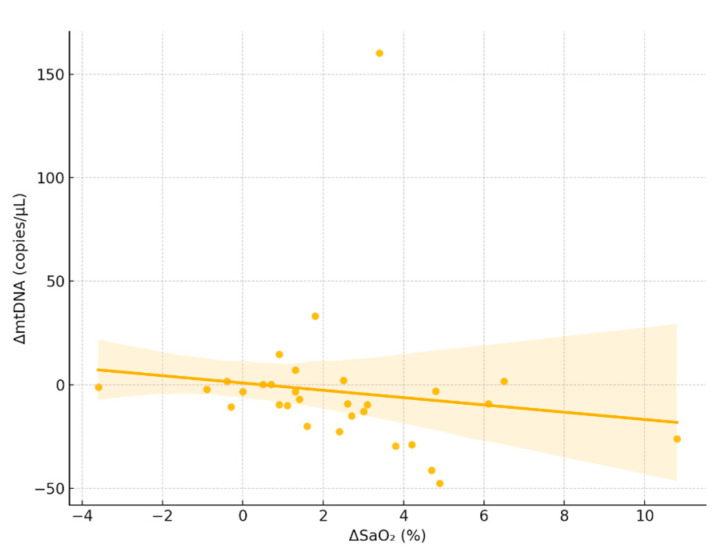
Scatterplot illustrating the relationship between intraoperative changes in arterial oxygen saturation (ΔSaO_2_) and changes in circulating mitochondrial DNA levels (ΔmtDNA). Each dot represents an individual patient (N = 30). A statistically significant inverse correlation was observed (Spearman *ρ* = –0.39, *p* = 0.032), suggesting that greater increases in oxygen saturation may be associated with reduced release or enhanced clearance of mtDNA. The shaded area represents the 95% confidence interval of the linear regression.

No significant associations were found between ΔmtDNA and changes in arterial blood gas parameters, including ΔPaO_2_ (*ρ* = –0.13, *p* = 0.48), ΔPaCO_2_ (*ρ* = 0.17, *p* = 0.36), and ΔHb (*ρ* = 0.08, *p* = 0.68), as determined by Spearman rank correlation. Only the inverse correlation with ΔSaO_2_ (*ρ* = –0.39, *p* = 0.032; Figure 2) reached statistical significance.

A non-significant trend was observed with respect to sex, indicating slightly higher ΔmtDNA levels in the male participants (median ΔmtDNA: –5.06 vs. –9.45; *p* = 0.182, Mann–Whitney U test). No correlations were found between ΔmtDNA and age (*ρ* = 0.17, *p* = 0.38) or ASA classification (*ρ* = –0.03, *p* = 0.89). Furthermore, there was no significant correlation between baseline mtDNA levels and sex, age, or ASA classification.

## 4. Discussion

This study investigated perioperative changes in circulating mtDNA levels in patients undergoing high-frequency jet ventilation during elective laryngotracheal surgery. Contrary to our initial hypothesis, mtDNA levels significantly decreased from spontaneous breathing to termination of jet ventilation.

This finding was unexpected. Previous studies suggested that mechanical ventilation, particularly when combined with high inspiratory oxygen fractions, as routinely applied during jet ventilation, can promote mitochondrial damage through oxidative stress and thereby increase mtDNA release [1,4,5,23]. The observed decline in mtDNA levels suggests that jet ventilation may have a less harmful effect on mitochondrial integrity than previously assumed. Jet ventilation, as a specific form of high-frequency, high-driving pressure ventilation, delivers small tidal volumes through an open airway system. These features, although potentially associated with non-physiological airflow dynamics, may reduce alveolar overdistension and shear forces. The present data may thus reflect a lung-protective effect of jet ventilation under specific conditions, though further research is required to substantiate this interpretation.

Alternative mechanisms may account for the reduction in circulating mtDNA. First, decreased plasma levels do not necessarily reflect reduced cellular release. It is possible that intraoperative physiological shifts enhance mtDNA clearance, redistribution, or sequestration. Circulating mtDNA is subject to enzymatic degradation by plasma DNase I, which may remain active, or even become upregulated, in response to systemic stress or procedural stimuli [24,25]. Moreover, mtDNA can be taken up by immune cells, encapsulated in extracellular vesicles, or compartmentalized in tissue, making it less detectable in plasma without implying reduced mitochondrial stress [9,26,27]. This could reflect a temporary shift in mtDNA kinetics caused by intraoperative changes in circulation or immune function.

A further possible explanation involves the activation of endogenous antioxidant mechanisms that help buffer oxidative stress. It is also conceivable that the specific delivery characteristics of jet ventilation prevent the buildup of oxidative burden to levels sufficient to trigger detectable mtDNA release.

Alternatively, the specific delivery characteristics may prevent the accumulation of oxidative stress to levels sufficient to trigger detectable mtDNA release. Supporting this, prior studies have shown that targeted mitochondrial repair mechanisms can mitigate hyperoxia-induced mtDNA damage, limiting apoptosis in alveolar epithelial cells [28,29]. In light of our findings, jet ventilation may exert less oxidative and mechanical stress than previously assumed.

Notably, no significant associations were observed between changes in mtDNA and the duration of ventilation, surgical procedure, or ASA classification. These findings suggest that the effect is not strictly time- or comorbidity-dependent within this cohort. However, the relatively limited sample size warrants cautious interpretation. The high proportion of ASA I patients in our cohort may be attributed to the predominance of vocal cord surgery for benign lesions, which are typically performed in otherwise healthy individuals and therefore not necessarily associated with significant comorbidities.

Patients were ventilated with a FiO_2_ of 0.8, a standard practice to maintain adequate oxygenation during open-airway ventilation. This was reflected in arterial blood gas measurements, which showed a marked increase in PaO_2_ from 123.5 mmHg at baseline to 222.4 mmHg at the end of jet ventilation. However, in jet ventilation, the Venturi effect entrains ambient air, resulting in mixing with the applied jet gas and thus reducing the oxygen fraction of 0.8 in the airways [12,30]. Previous research has demonstrated that high inspiratory oxygen fractions and hyperoxia induce oxidative stress and enhance the production of ROS, leading to mitochondrial membrane damage and disruption of mtDNA [6,7,20,23,31,32,33]. Surprisingly, despite the high FiO_2_ applied, we observed a significant decline in circulating mtDNA levels over the course of jet ventilation. This contrasts with the oxidative stress model and suggests that additional factors, such as open-airway dynamics or intraoperative regulation of immune and antioxidant responses, may modulate mtDNA release.

These findings underscore the complexity of interpreting mtDNA levels as direct markers of pulmonary cellular injury. High inspiratory oxygen fraction alone does not necessarily explain increased mitochondrial damage. This mismatch between oxygen levels and mtDNA suggests not only lower release but also altered clearance, degradation, or redistribution influenced by anesthesia or surgery-related factors. Therefore, further investigations are needed to explore the kinetics of mtDNA release and clearance in response to different ventilation strategies, ideally incorporating serial perioperative measurements and cell-type-specific analyses.

## 5. Limitations

This study has several limitations that warrant consideration. First, the sample size was relatively small, which limits the statistical power, particularly in subgroup analyses (e.g., by ASA classification or sex). As such, the findings should be interpreted with caution and regarded as hypothesis-generating. Second, only two timepoints for mtDNA measurement were available, making it impossible to capture dynamic changes or delayed responses that may occur beyond the immediate postoperative phase. Third, while mtDNA is increasingly used as a marker of cellular stress, it is not specific to lung tissue and may reflect systemic alterations influenced by anesthesia, immune activity, or plasma clearance mechanisms. Furthermore, the absence of a control group undergoing conventional ventilation precludes a direct comparison of mtDNA responses across ventilation modes.

To address the limitations of our current study, a follow-up investigation is planned. This future study will include a control group undergoing conventional volume-controlled ventilation, allowing a direct comparison of mitochondrial DNA dynamics across ventilation strategies. Additionally, we aim to include serial blood sampling at multiple perioperative timepoints to better capture the kinetics of mtDNA release and clearance.

## 6. Conclusions

The observed decline in circulating mtDNA during jet ventilation suggests a potentially lower mitochondrial stress response than expected and underscores the need for further investigation into ventilation-specific molecular effects.

## Figures and Tables

**Table 1:** Demographic characteristics of the study population.

Variables	Mean ± SD	Range	*n* (%)
Age (years)	48.8 ± 16.1	22–79	30
Height (cm)	174.2 ± 9.2	160–196	30
Weight (kg)	77.4 ± 12.0	58–101	30
BMI (kg/m^2^)	25.5 ± 3.2	19.8–31.2	30
Male			18 (60%)
Female			12 (40%)
ASA I			19 (63%)
ASA II			9 (30%)
ASA III			2 (7%)
*Type of surgery:*			
Vocal cord surgery			14 (47%)
Laser microsurgery			11 (37%)
Subglottic surgery			3 (10%)
Panendoscopy			1 (3%)
Supraglottic surgery			1 (3%)

N = 30; *n* (%). Demographic data are presented as mean ± standard deviation (SD) and range, where applicable. BMI = body mass index; ASA = American Society of Anesthesiologists physical status classification.

**Table 2:** Perioperative changes in circulating mtDNA and respiratory parameters.

Variable	Before Jet Ventilation—T0	After Jet Ventilation—T1	Mean Difference (95% CI)	*p*-Value
Circulating mtDNA (copies/µL) ^a^	18.0 ± 12.6	14.6 ± 30.8	−3.4 (−16.4–9.6)	0.009
PaO_2_ (mmHg) ^a^	123.5 ± 80.5	222.4 ± 86.9	+98.9 (53.5–144.3)	0.000
SaO_2_ (%)	96.8 ± 2.7	99.2 ± 1.2	2.4 (1.4–3.4)	0.000
PaCO_2_ (mmHg)	40.6 ± 5.7	39.2 ± 5.6	−1.4 (−4.1–1.3)	0.328
Hb (g/dL)	13.3 ± 1.3	12.3 ± 1.3	−1.0 (−1.2–−0.8)	0.000

T0 = pre-anesthesia baseline; T1 = post-jet ventilation; mtDNA = mitochondrial DNA; CI = confidence interval. Data are shown as mean ± standard deviation. Δ = difference T1−T0. ^a^ Data are presented as mean ± standard deviation. Mean differences and 95% confidence intervals (CIs) were calculated for each paired variable (T0 vs. T1). *p*-values were obtained using Wilcoxon signed-rank tests due to non-normal distributions.

## Data Availability

The data presented in this study are available on request from the corresponding author.

## Abbreviations

ASA, American Society of Anesthesiologists; DNase I, deoxyribonuclease I; FiO_2_, fraction of inspired oxygen; Hb, hemoglobin; mtDNA, mitochondrial DNA; PaCO_2_, arterial partial pressure of carbon dioxide; PaO_2_, arterial partial pressure of oxygen; PCR, polymerase chain reaction; ROS, reactive oxygen species; rt-PCR, real-time polymerase chain reaction; SaO_2_, arterial oxygen saturation; VILI, ventilator-induced lung injury.
